# The unfoldomics decade: an update on intrinsically disordered proteins

**DOI:** 10.1186/1471-2164-9-S2-S1

**Published:** 2008-09-16

**Authors:** A Keith Dunker, Christopher J Oldfield, Jingwei Meng, Pedro Romero, Jack Y Yang, Jessica Walton Chen, Vladimir Vacic, Zoran Obradovic, Vladimir N Uversky

**Affiliations:** 1 Center for Computational Biology and Bioinformatics, Indiana University Schools of Medicine and Informatics, Indianapolis, IN 46202, USA; 2 Center for Computational Biology and Bioinformatics, Indiana University School of Informatics, Indianapolis, IN 46202, USA; 3 Department of Biochemistry and Molecular Biology, Indiana University School of Medicine, Indianapolis, IN 46202, USA; 4 Center for Information Science and Technology, Temple University, Philadelphia, PA 19122, USA; 5 Center for Computational Biology and Bioinformatics, Department of Biochemistry and Molecular Biology, Indiana University School of Medicine, Indianapolis, IN 46202, USA; 6 Institute for Intrinsically Disordered Protein Research, Indiana University School of Medicine, Indianapolis, IN 46202, USA; 7 Institute for Biological Instrumentation, Russian Academy of Sciences, 142290 Pushchino, Moscow Region, Russia

## Abstract

**Background:**

Our first predictor of protein disorder was published just over a decade ago in the *Proceedings of the IEEE International Conference on Neural Networks *(Romero P, Obradovic Z, Kissinger C, Villafranca JE, Dunker AK (1997) Identifying disordered regions in proteins from amino acid sequence. Proceedings of the IEEE International Conference on Neural Networks, 1: 90–95). By now more than twenty other laboratory groups have joined the efforts to improve the prediction of protein disorder. While the various prediction methodologies used for protein intrinsic disorder resemble those methodologies used for secondary structure prediction, the two types of structures are entirely different. For example, the two structural classes have very different dynamic properties, with the irregular secondary structure class being much less mobile than the disorder class. The prediction of secondary structure has been useful. On the other hand, the prediction of intrinsic disorder has been revolutionary, leading to major modifications of the more than 100 year-old views relating protein structure and function. Experimentalists have been providing evidence over many decades that some proteins lack fixed structure or are disordered (or unfolded) under physiological conditions. In addition, experimentalists are also showing that, for many proteins, their functions depend on the unstructured rather than structured state; such results are in marked contrast to the greater than hundred year old views such as the lock and key hypothesis. Despite extensive data on many important examples, including disease-associated proteins, the importance of disorder for protein function has been largely ignored. Indeed, to our knowledge, current biochemistry books don't present even one acknowledged example of a disorder-dependent function, even though some reports of disorder-dependent functions are more than 50 years old. The results from genome-wide predictions of intrinsic disorder and the results from other bioinformatics studies of intrinsic disorder are demanding attention for these proteins.

**Results:**

Disorder prediction has been important for showing that the relatively few experimentally characterized examples are members of a very large collection of related disordered proteins that are wide-spread over all three domains of life. Many significant biological functions are now known to depend directly on, or are importantly associated with, the unfolded or partially folded state. Here our goal is to review the key discoveries and to weave these discoveries together to support novel approaches for understanding sequence-function relationships.

**Conclusion:**

Intrinsically disordered protein is common across the three domains of life, but especially common among the eukaryotic proteomes. Signaling sequences and sites of posttranslational modifications are frequently, or very likely most often, located within regions of intrinsic disorder. Disorder-to-order transitions are coupled with the adoption of different structures with different partners. Also, the flexibility of intrinsic disorder helps different disordered regions to bind to a common binding site on a common partner. Such capacity for binding diversity plays important roles in both protein-protein interaction networks and likely also in gene regulation networks. Such disorder-based signaling is further modulated in multicellular eukaryotes by alternative splicing, for which such splicing events map to regions of disorder much more often than to regions of structure. Associating alternative splicing with disorder rather than structure alleviates theoretical and experimentally observed problems associated with the folding of different length, isomeric amino acid sequences. The combination of disorder and alternative splicing is proposed to provide a mechanism for easily "trying out" different signaling pathways, thereby providing the mechanism for generating signaling diversity and enabling the evolution of cell differentiation and multicellularity. Finally, several recent small molecules of interest as potential drugs have been shown to act by blocking protein-protein interactions based on intrinsic disorder of one of the partners. Study of these examples has led to a new approach for drug discovery, and bioinformatics analysis of the human proteome suggests that various disease-associated proteins are very rich in such disorder-based drug discovery targets.

## Background

More than seventy years ago, it was speculated that antibody binding depends on unfolded rather than structured protein [1,2]. Specifically, Linus Pauling suggested that high flexibility enables one antibody molecule to bind to differently shaped antigens. The specific idea was that of conformational selection in which the flexible antibody would randomly fluctuate among the different structures, with binding by a particular antigen selecting the structure that fits from the other conformers among the ensemble [2]. The current body of evidence suggests that there are approximately two broad classes of antibodies, specific and non-specific. The sequence of a highly specific, high-affinity antibody folds into a specific structure that fits with its cognate antigen (with perhaps slight structural shifts of both the antibody and antigen). On the other hand, at least some of the low affinity, nonspecific antibodies contains binding sites that are disordered in isolation but become differently folded when bound to different partners. A recent assembly of structural data on antibody-antigen interactions supports the early conjectures cited above (manuscript in preparation).

More recently, involvement of intrinsic disorder in molecular recognition has been suggested to involve at least two possible mechanisms [3]. Conformational selection, as described by Pauling [2] and later by Karush [4], is the first mechanism. Alternatively, a local part of the binding region could form an interaction followed by concomitant binding and folding over the remainder of the interface [5-7]. While described sometime ago [6], this second mechanism was recently described in terms of folding funnel concepts and called the "fly casting mechanism" [7]. For two recently studied molecular recognition events, experimental evidence has been provided for the latter mechanism [8,9]. Both of these recently studied interfaces are fairly large and extend over significant lengths of the intrinsically disordered proteins. Mixed mechanisms of course are possible, with a subregion of the interface interacting via conformational selection, followed by concomitant binding and folding for the remainder of the interface. The choice between conformational selection and concomitant binding and folding might be due in significant degree to the overall size of the interaction surface.

The existence of unstructured, or incompletely structured, proteins under physiological conditions began to be reported almost sixty years ago, with several additional reports in the following decades [4,10-13]. Since the 1970s, an increasingly strong stream of disordered protein examples has been revealed, and many of these are described in our database of intrinsically disordered proteins [14,15]. This database also contains a bibliography that is showing explosive growth, especially over the last few years.

The crowded conditions inside the cell have been suggested to cause intrinsically disordered proteins to fold into 3D structure. To test this possibility, intrinsically disordered proteins were subjected to molecular crowding by adding high concentrations of agents such as glucose. Such *in vitro *molecular crowding experiments lead to successful folding of an acid-unfolded globular protein [16], but fail to induce folding in several intrinsically disordered proteins [16,17], suggesting perhaps that crowding leads to a deep energy well for a protein that folds under appropriate conditions but that crowding cannot induce a deep energy well for a protein with a sequence that is incommensurate with folding.

In-cell NMR experiments indicate that some proteins or protein regions remain unfolded even when crowding occurs inside a cell [18-20]. Another in-cell NMR report [21] involving some of the same authors was later retracted because protein leakage from the cells led to misleading data [22]. The earlier experiments [18,19] may not have suffered from the same leakage problems, which might have been specific for the protein used in the later studies [22]. Overall, these experiments provide additional evidence that intrinsically disordered proteins remain incompletely folded inside the cell, but additional experiments need to be carried out in order to increase confidence in these results.

A number of different terms have been used to describe these proteins, including rheomorphic [23], natively denatured [24], natively unfolded [25], intrinsically unstructured [26], and several variants of disordered [27-29]. By now, several reviews on these proteins have appeared [16,17,30-34]. We use "intrinsically disordered" to describe all types of incompletely folded proteins and regions, and we use "natively unfolded" or "intrinsically unstructured" to indicate random-coil-like and pre-molten globular forms. Collapsed random coils as recently described for polyQ [35,36] are similar to, if not identical with, the premolten globule form and in our view these structures fit into the "natively unfolded" category. However, there is not a consensus in this field regarding nomenclature, which suggests the need for a disordered protein ontology.

**Figure 1:**
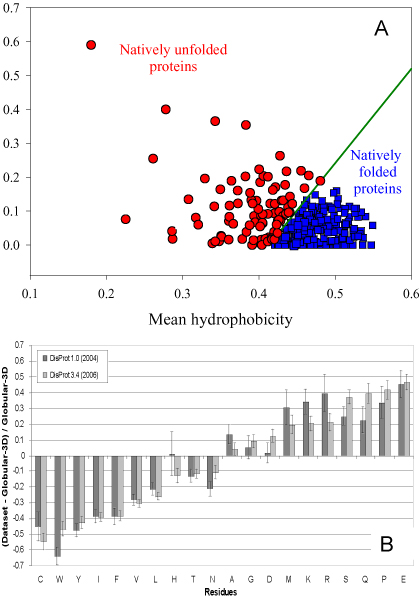
**Peculiarities of the amino acid sequences of intrinsically disordered proteins**. **A**. Mean net charge versus mean hydropathy plot (charge-hydropathy plot) for the set of 275 folded (blue squares) and 91 natively unfolded proteins (red circles) [37]. **B**. Amino-acid composition, relative to the set of globular proteins Globular-3D, of intrinsically disordered regions 10 residues or longer from the DisProt database. Dark gray indicates DisProt 1.0 (152 proteins), whereas light gray indicates DisProt 3.4 (460 proteins). Amino acid compositions were calculated per disordered regions and then averaged. The arrangement of the amino acids is by peak height for the DisProt 3.4 release. Confidence intervals were estimated using per-protein bootstrapping with 10,000 iterations [40].

Just as the amino acid sequence codes for protein structure, so might the sequence also code for lack of structure or disorder. Development of a predictor of protein disorder is one way to test the hypothesis that disorder is encoded by the amino acid sequence. Furthermore, study of disorder prediction provides a means to understand "the protein disorder code." For example, Figure 1A shows that "natively unfolded" proteins (a subset of intrinsically disordered proteins that have little or no ordered structure under physiologic conditions and behave as random coils or pre-molten globules [37-39]) are specifically localized within a unique region of charge-hydropathy phase space, indicating that a combination of low overall hydropathy and high net charge represent a unique structural feature of "natively unfolded" proteins [37]. In more general terms, certain amino acid residues have been found to be highly "order-promoting" (namely cysteine, tryptophan, tyrosine, isoleucine, phenylalanine, valine, leucine, histidine, threonine, and asparagine) while others are highly "disorder-promoting" (namely aspartic acid, methionine, lysine, arginine, serine, glutamine, proline, and glutamic acid) [40-42]. These order-inducing and disorder-inducing amino acid trends are further illustrated by Figure 1B, which depicts the relative amino acid compositions of intrinsically disordered regions available in the DisProt database [15,43] in comparison with a set of structured (or ordered) proteins [40]. In this case, these amino acid compositions were compared by means of a profiling approach [30,44].

During the fourth Critical Assessment of Structure Prediction (CASP) experiment, we worked with the meeting organizers to carry out disorder prediction on the various protein targets. This trial was deemed a success (C.J. Brown, unpublished), so disorder prediction was included in the subsequent CASP 5, 6, and 7 experiments [45-47]. Inclusion of disorder prediction in the CASP experiments has stimulated a rapid increase in the number of such predictors, with at least 25 different predictors having been developed by now. A collection of links to many of these is maintained at the Database of Disordered Protein website .

Several disordered protein predictors have been compared in recent publications [40,45-51]. As more disordered proteins have been identified, and as more sophisticated machine learning methods have been applied, the per residue prediction accuracy has risen from ~70% to ~85%. A likely-to-be significant impediment to further improvement is the misclassification of the residues in the training sets.

Application of the disorder predictors to various organisms in the three domains of life, namely, prokaryotes, archaea, and eukaryotes, reveals a large increase in disorder among the eukaryotes compared to the other two types of organisms [48,52,53]. One related speculation is that more disorder is needed for signaling and coordination among the various organelles in the more complex eukaryotic domain [54].

The recent explosion of papers on intrinsically disordered protein contains many new discoveries on these proteins by a large number of investigators. There is neither time nor space to adequately cover these important advances. Herein we focus mainly on our own work; and we hope that other researchers in this field will not be offended by this approach. In the following are seven short stories that briefly review recent research on disordered proteins published by our group. These include the following: (1) A bioinformatics study of the relationship between disorder and function in the Swiss Protein Database [55-57]; (2) An introduction of the molecular recognition feature (MoRF) concept and characterization of various MoRFs and MoRF-binding proteins [58-61]; (3) The mechanisms by which one disordered region can bind to many partners and by which many different disordered sequences can bind to one site on one protein partner [62,63] thereby contributing to the complex protein-protein interaction networks that are observed in nature; (4) The observation that regions of mRNA that undergo alternative splicing code for disordered protein much more often than they code for structured protein [64]; (5) A bioinformatics study on conservation of intrinsic disorder in protein domains and protein families [65,66]; (6) An introduction of the disordered proteins in disease (or D^2^) concept, which is based on bioinformatics analysis that indicate an abundance of intrinsic disorder in disease-related proteins [38,67-74]. (7) A novel method for drug discovery based on regions of disordered protein [75]. The novel drug discovery method suggests how the observations in the first six studies might be put to practical use.

## Intrinsic disorder and protein function

Our overall goal is to understand relationships between amino acid sequence and protein function so that, given a new sequence, possible functions could be suggested to interested experimentalists for laboratory testing. For proteins that form 3D structure, this is a well developed problem, but for intrinsically disordered proteins, work on this problem is just beginning. First we will very briefly review function prediction for structured proteins, and then we will compare and contrast the very limited amount of work in this area for intrinsically disordered proteins.

### Function prediction for structured proteins

For structured proteins, sequence homology, if obvious enough, can provide leads regarding protein function [76-78]. Attempts to improve sequence matching for function prediction have been carried out [79]. If no suggestive homologue can be found, an alternative approach is to determine the 3D structure and then to search structure for functional clues, such as residues positioned in space like the same or functionally similar residues in known active sites [80-82]. Often evolution within a family of related proteins can be helpful by means of the evolutionary trace approach [83]. Recent advances have been made in the assessment of binding sites using both structural and sequence homology [84]. For example, in order to create an automated annotation process involving the appropriate knowledge representation and prediction of functionally important residue environments, a method for extraction of features from sequence, sequence alignments, three-dimensional structure, and structural environment conservation in catalytic sites was recently proposed [85]. This tool was used to develop a model for automated identification of catalytic residues in unannotated protein structures. Application of this tool revealed that catalytic residues can be reliably predicted even for enzymes with new folds [85].

### Function prediction for disordered proteins

Our first efforts to associate disorder with function were carried out by manual literature searches. In the development of our protein disorder predictors, we wanted to use disorder characterized by methods other than missing coordinates in X-ray structures, especially to test whether disorder identified by different methods was different at the amino acid sequence level [86]. Therefore, we had accumulated manuscripts describing disordered proteins and regions of disorder characterized by a variety of methods such as NMR, circular dichroism, small angle X-ray scattering, and so on. In addition, we found many examples in which the disorder indicated by missing coordinates in X-ray crystal structures had been confirmed by other methods. Given these proteins and their associated manuscripts, we then carried out literature searches for functions associated with these well studied disordered protein examples. Out of more than 100 disordered proteins and regions, these manual searches identified 27 different functions, and at least one (and commonly more than one) of these functions was found to be associated with > 80% of the disordered proteins or regions. Of course when a given disordered region or protein has no associated function, it is unclear whether the given disordered protein has no function or whether the function of the given disordered protein has simply not yet been found [87,88].

Among the various functions found for disordered regions, even superficial analysis of "natively unfolded" proteins revealed that many of them undergo disorder-to-order transitions when stabilized by binding with specific targets [37]. In fact, for the majority of proteins described in that study, the existence of ligand-induced folding has been established. Examples include induced structure formation upon binding with DNA (or RNA) for protamines, Max protein, high mobility group proteins HMG-14 and HMG-17; osteonectine, SDRD protein, chromatogranins A and B, Δ131Δ fragment of SNase, and histone H1. Other examples include folding of cytochrome *c *in the presence of heme, folding of ostecalcine induced by cations, secondary structure formation in parathyroid hormone related protein induced by membrane association, structure formation in glucocorticoid receptor brought about by association with trimethylamine N-oxide, folding of histidine-rich protein II induced by heme; and structure formation and compaction of prothymosin-α mediated by zinc [37]. Therefore, among the major functions of these unstructured, intrinsically disordered proteins are nucleic acid binding, metal ion binding, heme binding and interaction with membrane bilayers [37].

For structured proteins, proteins can be grouped together if they display a common 3D fold as for example in the CATH [89] and SCOP [90] databases. Often these proteins with common folds have recognizable sequence similarity and so can be grouped into evolutionarily-related protein families. Sometimes, proteins have similar folds without recognizable sequence similarity [91].

Sequence matching can be used to group disordered proteins into related sets just as is done for structured proteins. Perhaps because of the absence of structural constraints, however, disordered proteins typically show higher rates of mutations than do structured proteins [88], so it is often more difficult to identify sequence relationships among disordered proteins. The functionally important residues within a disordered region tend to be a small percentage of the total number of residues and so their conservation tends to be obscured because of the mutations of surrounding residues. Given these limitations with regard to sequence matching for disordered proteins, we have tried to develop alternative strategies.

Our attempts to develop clustering algorithms for finding functionally related groups of disordered proteins have yet not been very successful. These failures encouraged us to develop an alternative approach based on predictions of disorder. For this approach, we randomly partitioned a set of disordered proteins into two subsets and developed separate predictors for each group. We then applied the two predictors to all the proteins, and repartitioned the proteins based on which predictor gave the more accurate results. We next retrained the two predictors using the two redistributed sets, and then repeated the competition and the redistribution. We carried out these steps iteratively until the assigned partitions converged. To test for reproducibility, we repeated the original random partition and then repeated the entire experiment several times. The two sets of proteins that resulted were mostly the same for the different initializations, suggesting that the overall approach gives a reproducible partition of the disordered proteins into two groups [92].

Next, we repeated the overall process, but with three subsets, four subsets, five subsets and six subsets instead of two as tried originally. If this approach gives meaningful results, improved agreement between disorder prediction and observation would be expected due to increased homogeneity within each subset. Prediction did improve for the two and three subset partitioning, but not for four, five nor six subset partitioning. We called the three subsets of disordered proteins "flavors." The three subset flavors were labeled V, C and S [92].

The functions associated with the various proteins in each subset were then determined by literature searches. While the different functions did not separate completely among the different subsets, some of these flavors showed a greater tendency to display particular functions, *e.g. *S was associated with protein binding, V was associated with RNA binding, and C was associated with posttranslational modification sites [92].

More work on this approach might lead to improved understanding of the relationships between sequence and function for disordered proteins. To make the original study more manageable, several simplifications were carried out, and these simplifications likely diminished the ability to discriminate different flavors of disorder. Removing these simplifications might enable prediction of function from sequence for at least some regions of disorder. Indeed, using an approach more standard than ours, an important and remarkable success has recently been achieved in the prediction of function from sequence for disordered proteins [93].

More recently we carried out an analysis of the functional annotation over the entire Swiss Protein database from a structured-versus-disordered point of view [55-57]. The first step was to find keywords associated with 20 or more proteins in SwissProt. For each keyword-associated set, one thousand length-matching and number-matching sets of random proteins were drawn from Swiss Prot. Order-disorder predictions were carried out for the keyword-associated sets and for the matching random sets. If a function described by a given keyword were carried out by a long region of disordered protein, one would expect the keyword-associated set to have a greater amount of predicted disorder compared to the matching random sets. The keyword-associated set would be expected to have less prediction of disorder compared to the random sets if the keyword-associated function were carried out by structured protein. Given the predictions for the function-associated and matching random sets, it is possible to calculate the p-values, where a p-value > 0.95 suggests a disorder-associated function, a p-value < 0.05 suggests an order-associated function, and intermediate p-values are ambiguous.

Out of 710 keywords each being assigned to at least 20 proteins, 310 had p-values < 0.05, suggesting order-associated functions, 238 had p-values > 0.95, suggesting disorder-associated functions, and the remainder, 170, gave intermediate p-values, yielding ambiguity in the likely function-structure associations [55-57].

**Figure 2:**
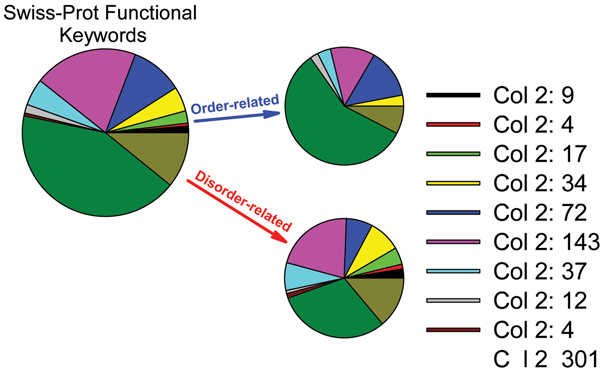
Functional anthology of intrinsic disorder.

When the functional keywords were partitioned into eleven functional categories (Biological processes, cellular components, developmental stage, etc.) order-associated keywords were found for seven of the categories, but disorder-associated keywords were found for all eleven categories [55]. This observation supports a previous conjecture that the functional repertoire is larger for disordered proteins compared to that for structured proteins [28]. Figure 2 represents summary of this analysis showing relative distributions of these eleven functional categories among intrinsically disordered and ordered proteins.

Considering the biological processes category, the order-associated keywords nearly all described processes carried out by (necessarily structured) enzymes (examples: amino acid biosynthesis, purine biosynthesis, lipid synthesis, etc) or by (necessarily structured) integral membrane proteins (electron transport, sugar transport, ion transport). On the other hand, in this same category, the disorder-associated keywords described processes that typically involve control or regulation (differentiation, transcription, cell cycle, growth regulation, etc.). These observations slightly broaden an earlier conjecture that structured proteins are primarily associated with catalysis while disordered proteins are associated with signaling and regulation [28,94].

Finally, it is interesting to compare the individual keywords associated with disorder prediction and with those associated with the absence of disorder prediction (which indicate structure-associated functions). Ribonucleoprotein and ribosomal protein are two disorder-associated keywords with the highest Z-scores (values of 22.1 and 20.6, respectively). Interestingly, the Z-scores drop off to values less than 10 after just a few proteins. Oxidoreductase and transferase are the order-associated keywords with the highest Z-scores (values of -29.5 and -24.5, respectively). Furthermore, the drop-off to values less than 10 occurs more slowly for the order-associated keywords. One possible explanation is that the structured regions for most of the proteins comprise most of the amino acid sequence for the given protein whereas the disordered region might comprise a small part of the entire sequence.

Another interesting feature of these data is that the top 20 order-associated keywords all end in "ase," indicating that all are enzymes of one type or another. This suggests that, for the order-associated keywords, the overall approach works rather well. Although some laboratory genetic engineering experiments have yielded molten globules with enzymatic activity [95], to our knowledge currently known natural enzymes are structured proteins.

Further studies on the disorder-associated key words involved ranking the proteins in each category by Z-score and then carrying out manual literature searches for evidence of association between disorder and function for the highest-ranking proteins. Indeed, for a significant fraction of the high Z-score proteins with functions predicted to be associated with disorder, an association between disorder and function was confirmed by these manual literature searches [56,57].

The tedious work of confirming the associations between disorder and function needs to be carried out for more of the protein groups in this study. It would then be interesting to study these groups of proteins by the methods described above or by new methods to find sequence-function relationships for disorder-associated functions. Such work would provide the basis for enabling researchers to infer (disorder-associated as well as order-associated) function from sequence.

## Intrinsically disordered proteins as interactors: MoRFs, linear motifs, "preformed elements" and "fuzzy complexes"

Protein-protein and protein-nucleic acid interactions, being central to many processes in molecular biology, often involve coupled folding and binding of at least one of the partners and sometimes involve coupled binding and folding for both partners. When a protein-protein interaction involves an intrinsically disordered partner, the methods developed for predicting protein-protein interactions based on known structures are simply not applicable. For intrinsically disordered proteins, new methods and new approaches are needed. The importance of predicting regions of disordered proteins that bind to partners of course depends on the commonness of such proteins.

### Finding MoRFs

**Figure 3:**
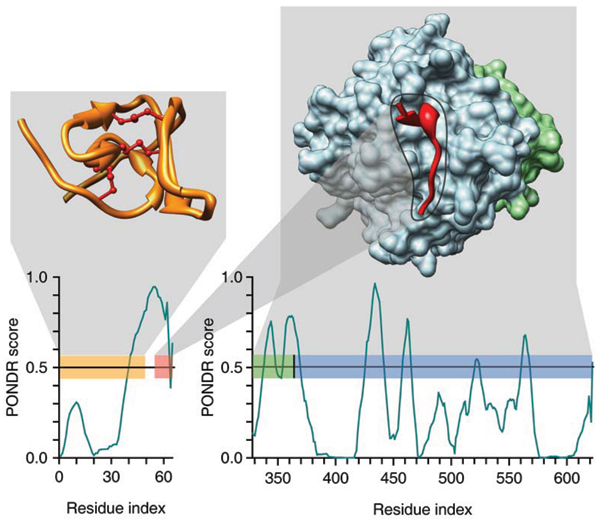
**PONDR-based analysis of hirudin and thrombin**. The correspondence of PONDR^® ^VL-XT predictions and regions of known structure are shown. Two PDB structures are presented – 5HIR (left) and 1NO9 (right) – where each chain is color coded – folded N-terminal domain of hirudin (yellow, disulphide bridges are shown by maroon lines), acidic C-terminal domain of hirudin (red) bound to a heavy chain of thrombin (blue), and light chain of thrombin (green). These color codes are also used for bars in two PONDR^® ^VL-XT plots – (top) hirudin and (bottom) thrombin – to indicate the positions of the regions of known structure in the context of the PONDR^® ^VL-XT predictions. Drawn over these bars, hash marks show the residues in contact with other chains, where the color of the hash mark corresponds to the color code of the chain in contact. Black hash mark in the PONDR^® ^VL-XT plot for thrombin corresponds to the factor Xa cleavage site. A predicted α-MoRF region of hirudin is shown in corresponding PONDR^® ^VL-XT plot as a pink bar.

We noticed several particular examples in which binding sites within disordered regions coincided with dips in our disorder prediction plots, especially PONDR VL-XT plots [96], so we developed a predictor of binding sites within disordered regions based on disorder prediction [58]. We suggested that these segments contain molecular recognition features or MoRFs. This feature consists of a short region (on the order of 20 residues) that undergoes a disorder-to-order transition that is stabilized by binding to its partner; this short region is within a segment of disorder. These MoRFs were proposed to function in the recognition of protein or nucleic acid partners [58]. Figure 3 shows that a region of hirudin involved in interaction with thrombin has a peculiar and well-recognizable pattern, where short region of predicted order is surrounded by extended regions predicted disorder [73]. This specific pattern was used to develop a unique bioinformatics tool dedicated to the identification of potential protein-protein sites in intrinsically disordered proteins, namely the α-MoRF identifier [58]. The application of this identifier to various protein datasets revealed that the frequency of α-MoRFs in various types of proteins is highest in those associated with signaling and lowest in the metabolic enzymes. Evidently, these elements have advantages for cell signalling, e.g., allowing among others the decoupling of specificity/affinity, which provides a mechanism by which the strength and duration of signaling events can evolve separately [58].

This first α-MoRF identifier was developed using a training data set of a limited size (a set of 13 proteins containing 15 potential α-MoRFs). All the training examples were correctly identified by the algorithm, suggesting the possibility of overfitting. Recently, the prediction algorithms was improved by (1) including additional α-MoRF examples and their cross species homologues in the positive training set, (2) carefully extracting monomer structure chains from the Protein Data Bank (PDB) as the negative training set, (3) including attributes from recently developed disorder predictors, secondary structure predictions, and amino acid indices, and (4) constructing neural network based predictors and performing validation [61]. The sensitivity, specificity, and accuracy of the resulting predictor, α-MoRF-PredII, were 0.87 ± 0.10, 0.87 ± 0.11, and 0.87 ± 0.08 over 10 cross-validations, respectively [61].

### Linear motifs

A completely different approach for finding protein-protein interaction sites is to search for the few, function-associated residues that remain conserved in the sea of changes among the surrounding disordered regions. Such conserved residues have been called Eukaryotic Linear Motifs (ELMs) and methods for their discovery from sequence, analogous to finding transcription factor binding sites, have been developed [97-99]. The overall idea is to search for overabundance of particular residues in regions of sequence that lie outside of Pfam domains. The sets of sequences to be tested typically bind to one specific partner. Thus, evidently the conserved residues represent a binding motif within a linker between (Pfam) structured domains or in a disordered tail at the carboxy or amino terminus of a (Pfam) structured domain [99].

Currently, when the search is carried out for ELMs, Pfam domains are excluded. This exclusion typically results in increased focus on regions of intrinsic disorder. However, some Pfam domains contain regions predicted to be disordered with a high degree of conservation [65]. Furthermore, these disordered regions are often implicated in biological functions [66], thus giving a set of disorder-associated functional regions that are not considered by the current ELM analysis. Extending the current ELM analysis to include these Pfam-associated regions of disorder should be done.

ELMs are identified by their over-representation among protein sequences that bind to a common partner [97-99]. Short linear motifs (SLiMs) are also identified as specific sequence patterns that are over-represented in proteins that bind to a common partner, but the algorithms used to discover SLiMs employ filters to remove homologous proteins whereas the ELM-discovery algorithms do not [100]. Thus, ELMs and SLiMs are both identified as sequence patterns in multiple proteins that bind to a common target, with the SLiM-containing set likely to be entirely nonhomologous but with no such restriction on the ELM-containing set.

MoRFs differ from ELMs and SLiMs in not depending on a specific sequence motif, but rather upon a pattern in a disorder prediction output. Yet, interestingly, recent analysis suggests that linear motifs (LMs) (thus not differentiating between ELMs and SLiMs) show high overlap with MoRFs [101]. Taken all together, these observations suggest that regions of intrinsic disorder often play a role in protein-protein interactions. In addition, there are numerous documented cases where the binding of these disordered regions is coupled to their folding (reviewed in [102]).

### Discriminative features of MoRFs and their binding partners

Experimentalists have successfully used our MoRF predictors to discover sites of protein-protein interactions that were subsequently confirmed in laboratory experiments [103,104], and other studies independently verified the predicted interactions [105]. Application of this algorithm to databases of genomics and functionally annotated proteins indicates that α-MoRFs are likely to play important roles protein-protein interactions involved in signaling events. In agreement with this model, recent computational studies of such binding showed that the disordered partner might contain a "conformational preference" for the structure it will take upon binding, and that these so-called "preformed elements" tend to be helices [58-60,106]. An important output of induced folding is that this coupled binding and folding determines a unique combination of high specificity and low affinity [107] typical of the signaling and regulation interactions. More recent studies show that at least some disordered regions display template-dependent folding rather than preformed elements (see below, [62,63]).

**Figure 4:**
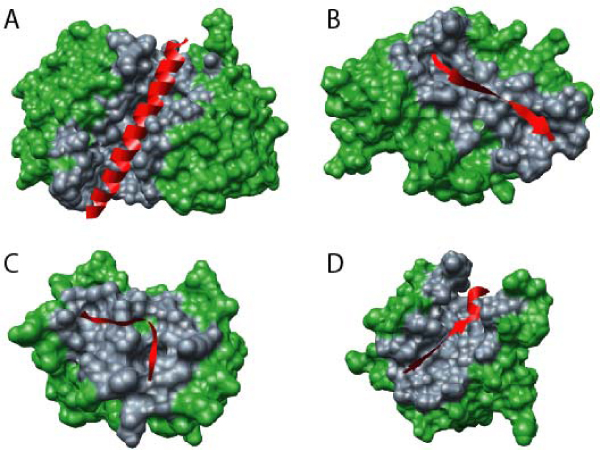
**Examples of structurally divergent MoRFs**. MoRFs (red ribbons) and partners (green surface) are shown (**A**) An α-MoRF, Proteinase Inhibitor IA3, bound to Proteinase A (PDB entry 1DP5). (**B**) A β-MoRF, viral protein pVIc, bound to Human Adenovirus 2 Proteinase (PDB entry 1AVP). (**C**) An ι-MoRF, Amphiphysin, bound to α-adaptin C (PDB entry 1KY7). (**D**) A complex-MoRF, β-amyloid precursor protein (βAPP), bound to the PTB domain of the neuron specific protein X11 (PDB entry 1X11). Partner interfaces (gray surface) are also indicated.

A search of PDB has revealed more than 2,500 short regions of one protein (MoRFs) associated with a globular domain of a second protein. Many of these short regions are related to each other, so the number reduces to several hundred families when they are grouped by sequence similarity. Most of these interactions are associated with signaling or regulation [59]. This PDB analysis revealed that MoRFs can be divided into three subtypes according to their structures in the bound state: α-MoRFs form α-helices, β-MoRFs form β-strands, and ι-MoRFs form structures without a regular pattern of backbone hydrogen bonds [59,60]. We have also found numerous complex MoRFs, which represent mixtures of these three structural forms. Illustrative examples of structurally divergent MoRFs are shown in Figure 4.

Although only a few MoRFs have been studied experimentally, our bioinformatics analysis suggests that all MoRFs are intrinsically disordered in the absence of their binding partners. This was done using the criteria of Gunasekaran *et al*. [108], who showed that the complexes of intrinsically disordered proteins have much larger interface and surface areas than those of complexes formed by pairs of structured proteins. In other words, Gunasekaran *et al*. have demonstrated that intrinsic disorder in the unbound state is reflected in the structures of the bound state through relatively large surface and interface areas.

**Figure 5:**
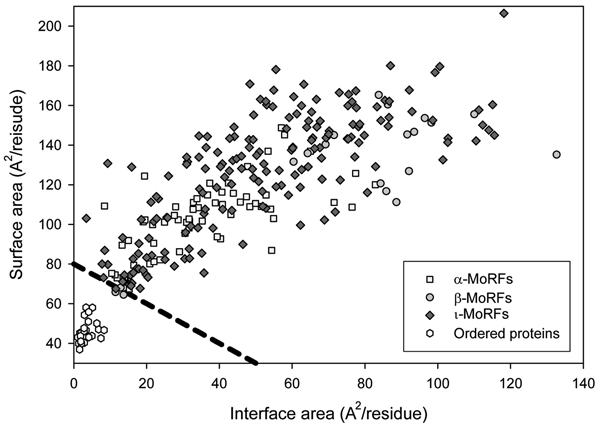
**Bioinformatics evidence for the unstructured character of MoRFs in their unbound states**. Surface and interface area normalized by the number of residues in each chain for MoRF and the OC datasets.

Using the approach and control datasets described above [108], a structural analysis of the bound structures of MoRFs in our dataset of 62 α-, 20 β- and 176 ι-MoRF was carried out (Figure 5). Almost all MoRFs in the dataset gave positions above the order-disorder boundary suggested previously, which indicates that these regions are likely to be disordered in isolation, while all ordered proteins gave positions below this boundary, which indicates these proteins are likely to be ordered in isolation [59].

Next, we examined several geometric and physiochemical criteria of MoRF-partner complexes [60]. The comparison of the compositions and physiochemical properties of MoRF and MoRF partner interface residues with the interface residues of homodimers, heterodimers, and antigen-antibody complexes indicated that there are significant differences in residue composition and several geometric and physicochemical properties that can be used to discriminate, with a high degree of accuracy, between various interfaces in protein interaction datasets [60].

**Figure 6:**
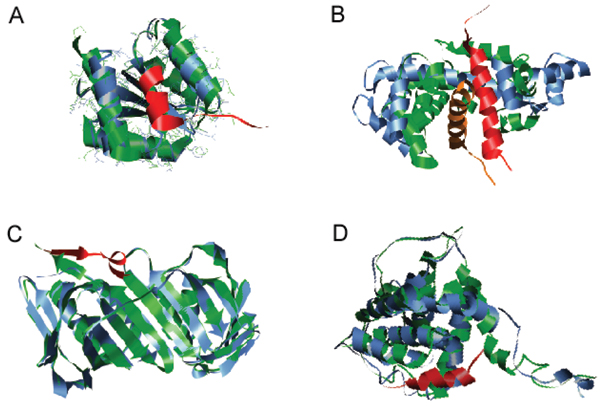
**Structural changes in MoRF partners**. Ribbon representation MoRF partners shown unbound (blue ribbons) and bound (green ribbons) to MoRFs (red ribbons). (**A**) Small scale structural alterations in CheY induced by binding of the MoRF region of FliM (PDB entries: unbound – 1U8T and bound – 1F4V). (**B**) Large scale structural alterations in calmodulin induced by binding to the MoRF of GAD (PDB entries: unbound – 1CLL and bound – 1NWD). (**C**) Partial disorder-to-order transition in PCNA induced by binding to the MoRF of FEN-1 (PDB entries: unbound – 1RWZ and bound – 1RXZ). (**D**) Partial order-to-disorder transition in Bcl-xL induced by binding to the MoRF of Bim (PDB entries: unbound – 1PQ0 and bound – 1PQ1).

The MoRF-partner complex formation was shown to be accompanied not only by the binding-induced folding of MoRFs, but also by noticeable structural changes in the MoRF partners which vary widely, from small scale movements to large scale movements and from partial folding to partial unfolding [60]. Figure 6 represents several illustrative examples of structural changes induced in MoRF partners by the complex formation. We did not find a single complex that was not accompanied by some structural adjustments in a MoRF partner induced by complex formation [60].

### "Fuzzy complexes"

Recently, it was suggested that intrinsically disordered proteins are able to become ordered following binding to their partner(s) and that this 'restores' the primacy of the classical structure-function paradigm according to which protein function is equated with a well-defined 3D structure [109]. However, the careful analysis of structures of many protein complexes in PDB revealed that this statement is not generally true. In fact, even in the crystal structures, the part(s) of the complexes that contribute productively to binding and function are structurally ill-defined, and cannot be described by a single conformational state, in other words demonstrating a significant amount of structural disorder or polymorphism in protein complexes [109]. According to the authors of this study, such disorder can be grouped into four mechanistic categories, where the crucial protein component might adopt a few or multiple alternative conformations ('polymorphic' model), or it might remain disordered but connect ('clamp' model) or neighbour ('flanking' model) ordered binding region(s). At the extreme, the majority or the whole of the bound IDP might remain disordered ('random' model). These observations paved the ground for the "fuzziness" phenomenon according to which functional disorder in protein-protein complexes is widespread covering a continuous spectrum of structural states from static to dynamic disorder and from segmental to full disorder [109]. It has been also argued that fuzziness in protein-protein interactions is beneficial in a variety of functional settings. Its existence, however, has thus far been largely overlooked because of the bias in our experimental approaches to obtain well-defined structures of complexes, and also with respect to our understanding of the functional relevance of such states [109].

### Disordered signalling conduits

A four-step disordered signalling conduit has been recently proposed to explain the functionality of the cyclin-dependent kinase inhibitor p27^Kip1 ^(p27) [9]. p27 is a small intrinsically unstructured protein [110] regulating cell proliferation through interactions with cyclin-dependent kinases (Cdks) [111]. A critical step in the G_1 _to S phase transition of cell division is the phosphorylation-dependent removal of the inhibitory p27 molecule from the surface of the cyclin A/Cdk2 complex. The initial level of p27 in the G_1 _phase is high, and this high level blocks the progression from G_1 _to S phase via the inhibition of Cdk2/cyclin A and Cdk2/cyclin E [112,113]. Therefore, the level of p27 has to decrease significantly for the Cdk2/cyclin complexes to become fully activated and for cell division to progress. The p27 level is controlled via translational regulation and ubiquitination-dependent proteolysis [114,115]. Ubiquitination of p27 at the G_1_/S transition is regulated by its phosphorylation [116]. In addition, abnormal ubiquitination-mediated degradation of p27 is common in human tumors [117].

Recently, a thorough study combined both biophysical and computational tools to reveal a complex four-step conduit model explaining the phosphorylation-mediated dislocation of p27 from cyclinA/Cdk2 [9]. In this model, the starting point is a ternary complex formed between p27 and the cyclin A/Cdk2 heterodimer. In the first step, Y_88 _of p27^Kip1 ^becomes phosphorylated by a non-receptor tyrosine kinase (NRTK), thereby making the Cdk2 active site accessible and leading to the Cdk2-controlled phosphorylation of p27 at T_187_. This latter phosphorylation promotes ubiquitination of p27 by the SCF^Skp2 ^E_3 _lyase complex. Finally, ubiquitinated p27 is degraded by the 26S proteasome, activating the cyclin A/Cdk2 complex and promoting the G1 to S phase transition [9].

The flexibility and lack of structure are key features for this p27 signaling conduit. The p27 chain encircles the cyclin A/Cdk2 complex and interacts with surface features at several well separated locations [118]. The lack of buried surface area within a single p27 chain in the complex further supports a fully disordered state for unbound p27. Such lack of internal structure facilitates the unzippering of complexes, which allows part of a complex to separate while maintaining other interactions [3]. In the case of p27, the flexibility allows part of the protein to separate from the surface of the ternary complex while many of the contacts remain intact. The flexibility of the tethered but otherwise free disordered segment further enables p27 to fold back, thereby accelerating phosphorylation via a unimolecular mechanism. The lack of structure also likely facilitates entry of p27 into the proteasome cavity where digestion occurs.

One of the possible explanations for the very complex and highly coordinated four-step signal conduit is that the biology for this particular example requires the disruption of an already-formed complex rather than the inhibition of complex formation [119]. Because of flexibility, the disruption of the p27/cyclin A/Cdk2 complex can proceed in a stepwise, segmental fashion, which likely provides a kinetic advantage for the overall process. By this approach, the interactions are dispersed bit-by-bit rather than all at once [119].

Furthermore, intrinsic disorder in the p27 conduit provides several general features that are useful for signalling interactions such as binding diversity, large interaction surface and uncoupled specificity and affinity. In fact, because a significant part of the binding energy has to be spent to fold a flexible protein, lack of structure and flexibility in the unbound state provide interactions with a mechanism to have both high specificity and low affinity, with the low affinity providing the basis for easy reversibility. On the other hand, a large interaction surface coupled with a high flexibility allows segmental association and dissociation, thereby providing additional opportunities for regulation and control as demonstrated in the conduit model [119].

## Intrinsic disorder and protein-protein interaction networks

Networks linking protein-protein interactions typically involve a few proteins binding to many partners (called hub protein or hubs) and many proteins interacting with just a few partners. How these networks acquired their architecture and how they evolved are both very active areas of research [120-122]. A News and Views article [123], which was longer than the article [124] it discussed, raised the possibility that the ability of hub proteins to bind to many partners might depend on new principles. In essence, the News and Views article raised the question: what feature of protein structure enables binding diversity?

Pauling's 70-year old conjecture that unfolded, dynamic protein ensembles could contribute to binding diversity provided the beginning for the present article. Since Pauling's initial work, several additional researchers have suggested that lack of structure (e.g. disorder) could contribute to the ability of a protein to bind to multiple partners, with several of these researchers providing experimental data in support of this concept [3,69,125,126].

To test the roles of disorder in the specific case of protein-protein interaction networks, we first collected a set of structurally characterized hub proteins [127]. Several hub proteins were found to be entirely disordered, from one end to the other, and yet to be capable of binding large numbers of partners. Other hubs contained both ordered and disordered regions. For these hubs, many, but not all, of the interactions mapped to the regions of disorder. Two highly structured hubs were found. For both of these structured hubs, 14-3-3 and calmodulin, the binding regions of their partner proteins were found to be intrinsically disordered [128,129]. However, it has proven very difficult to globally test whether structured hubs bind to disordered partners. A difficulty with such studies is that the partners often contain both order and disorder, and the disordered regions typically comprise only small fractions of the partner sequences. Thus, without knowing the binding region of each partner, it is difficult to estimate whether or not disorder is involved in any particular interaction.

Overall, our initial study suggested two primary mechanisms by which disorder is utilized in protein-protein interaction networks, namely one disordered region binding to many partners and many disordered region binding to one partner. Several groups have tested these overall ideas further via bioinformatics studies on collections of hub proteins, and these studies support the common use of disordered regions by hub proteins to bind to multiple partners [130-134]. These bioinformatics studies include further refinement of the analysis with the suggestion that disorder is very commonly used for regions that bind sequentially to multiple partners (so called "date hubs" [134]).

Without specific regard to protein-protein interaction networks, several years ago we considered possible roles of disorder in protein interactions. In that study, we proposed that "one-to-many" signaling be used to describe the capacity of one disordered region to bind to many partners. We further suggested that "many-to-one" signaling be used to describe how flexibility could enable multiple disordered regions to bind to one site on one partner [3]. While numerous papers suggest that flexibility could enable one protein to bind to many partners, we might have been the first to suggest that flexibility would provide a means for multiple sequences to bind to a common partner.

Recently we studied the detailed structures for a one-to-many example (namely, p53 using its disordered regions to bind to many partners), and we also studied the structures of a many-to-one example (namely, 14-3-3 using its single binding site to associate with many different disordered partners having different amino acid sequences).

Using a collection of structures currently available in the PDB, a single disordered region of p53 is observed to form a helix, a sheet, and two different irregular structures when binding to four different partners, respectively. The set of residues involved in these one-to-many interactions have an identical core set with slightly different extents on either side [62].

The accessible surface area (ASA) with regard to the solvent molecules can be calculated from the three dimensional structure of a protein analytically [135] or numerically [136]. The amount of ASA becoming inaccessible upon complex formation is likewise easily estimated [137] and can be presented as the ΔASA.

**Figure 7:**
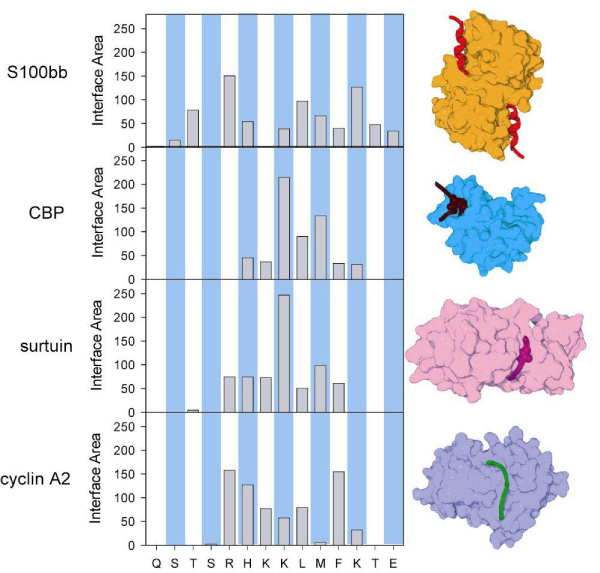
**Sequence and structure comparison for the four overlapping complexes in the C-terminus of p53**. (A) Primary, secondary, and quaternary structure of p53 complexes. (B) The ΔASA for rigid association between the components of complexes for each residue in the relevant sequence region of p53. The two hatched bars indicate acetylated lysine residues. Histogram of conserved predicted disorder effective length classes by kingdom.

Plotting the ΔASA versus the sequence position gives a binding profile (Figure 7). Interestingly, the single region of p53 bound to four different partners gives completely different binding profiles. For this example, the different partners "read" the same sequence in entirely different ways [62].

For a partner-binding disordered region, the binding profile is highly localized. In contrast, for a partner-binding structured protein, the binding profile comprises two (or more) localized regions that are considerably separated along the sequence. The separation results from bringing together different regions to form the active site in the structured protein. The DNA binding domain of p53 also has a complex, distinctive binding profile that is different from the profiles when binding to p53BP1 and p53BP2 [62]. The p53BP1 and p53BP2 profiles have similar p53 sequence localizations, but the detailed shapes of the profiles are quite distinctive.

Using structures currently in the PDB), five disordered sequences associated within a single binding groove in 14-3-3 provide an example of many-to-one signaling interactions. As suggested previously [3] the flexibility plays a major role enabling different sequences to fit into one binding site. Both backbone and side-chain flexibilities are needed to accomplish the structural shifts needed for the different sequences to fit into the common binding site [62].

Our earlier publication failed to consider the flexibility on the structured side of the complex (e.g. the flexibility in 14-3-3) for many-to-one signaling interactions. In this example, the structured protein side of the complex also uses flexibility to accommodate the binding of the many disordered segments to a common binding site.

The now famous induced-fit hypothesis was first proposed in 1958 [138]. In Koshland's original publication, a thought-experiment involving different amino acid side chains binding to a common site was described. For the different sequences to bind, an "induced fit" was suggested to be required in order to accommodate the different structures of the different side chains. The current text-book examples of induced fit describe an entirely different type of binding in which fairly rigid domains shift upon binding to their ligand. However, 14-3-3 binding to multiple peptides of different sequences is evidently the first example that closely corresponds to Koshland's original induced fit hypothesis. Comparing the interactions of 14-3-3 with the different peptide sequences confirms Koshland's original induced fit hypothesis, and these comparisons provide insight regarding the degree of structural change upon binding (manuscript in preparation).

## Intrinsic disorder and alternative splicing

Two or more mature mRNAs are produced from a single precursor pre-mRNA by the inclusion and omission of different segments in a process called "alternative splicing" [139,140]. The "exons" are joined to form the mRNA and the "introns" are left out [141]. So far alternative splicing has been commonly observed only in multicellular eukaryotes [142]. For humans and other mammals, 40 – 60% the genes yield proteins via the alternative splicing mechanism [143-145], and multiple proteins are often produced from a single gene. Alternative splicing very likely provides an important mechanism for enhancing protein diversity in multicellular eukaryotes [146].

Alternative splicing has affects on a diversity of protein functions such as protein-protein interactions, ligand binding, and enzymatic activity [147-149]. Therefore it comes as no surprise that abnormal alternative splicing has been associated with numerous human diseases, examples being myotonic dystrophy [150], Axoospermia [151], Alzheimer's [152] and cancer [153].

Alternative splicing that maps to protein structure would often lead to dysfunctional protein folding, most often causing loss of function. In some cases, however, the alternatively spliced structured protein can maintain function, albeit typically with a reduction in activity.

For alternative splicing that maps to structure, the alterations are generally of small size, are usually located on the protein surface, and are most often located in coil regions [154]. These features support efforts to predict the affects of alternative splicing on protein structure (and function) by homology modeling [155] and by a more sophisticated structural modeling and analysis [154]. Given the small sizes and locations of the changes resulting from alternative splicing, the different splice variants were predicted to fold into the same overall structures, with only slight structural perturbations that could be functionally important.

The structural implications given above are interesting, but only a small fraction of alternative splicing events have been mapped to structured proteins. Given that 40% to 60% of mammalian (human) genes are estimated to undergo alternative splicing, and given that there are several thousand mammalian proteins in PDB [156], we would expect to find several thousand examples to study. So far, however, despite exhaustive searches of PDB, only 20 examples have been reported [154]. Given the failure to find a significant number of examples of alternative splicing that map to regions of structure, what is the alternative?

To further understand the relationship between alternative splicing and structure we searched for alternatively spliced isozyme pairs and were able to find just five such pairs with structures determined for both partners [157-161]. Consistent with the modeling paper results discussed above [154], the folding of the protein isoforms pairs was nearly identical. The lack of significant structural perturbations occurred because alternatively spliced segments were either short regions on the surface of the structure (for two pairs) or were disordered regions (for the remaining three pairs). With regard to the two spliced structured segments, the larger structural perturbation corresponded to the omission of a short helix in the shorter splice variant. This omission led to a slight rearrangement of the neighboring secondary structure elements adjusted to accommodate the lack of the very short intervening helix. As for the three pairs for which the alternative splicing mapped to disordered regions, this suggests a possible explanation for the missing examples of splicing that map to the structures in the PDB.

Given the above data, we hypothesized that the protein folding problems discussed above would be solved for different isoforms if the alternatively spliced regions of mRNA were to code for regions of intrinsically disordered protein. If alternative splicing were to map to disordered regions, both multiple and long splice variants would be allowed because structural perturbation would not be a problem.

To test whether alternative splicing is associated with disorder, we built a collection of human proteins with structurally characterized regions of both structure and disorder. Next, we searched for data on alternative splicing for all of these proteins. At that time we were able to find just 46 human proteins with 75 alternatively spliced segments all of which were located in structurally characterized regions [64].

**Figure 8:**
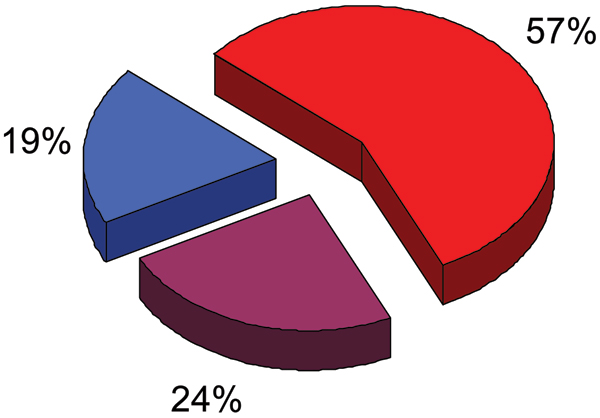
**Abundance of intrinsic disorder in alternatively spliced regions**. Fractions of alternatively spliced regions of RNA coded for entirely disordered protein, for both ordered and disordered protein, and for fully structured regions are shown as red, violet and blue pieces of the pie chart respectively.

Figure 8 shows that of these 75 alternatively spliced regions of RNA, 43 (57%) coded for entirely disordered protein, 18 (24%) coded for both ordered and disordered protein (with the splice boundaries very often in, or very near to, the disordered regions), and just 14 (19%) coded for fully structured regions [64].

While small in number, these 43 disorder-associated alternatively spliced regions and 18 mixed-structure regions are significantly larger than the 14 regions associated with regions of structure. Nevertheless, it would be very useful to enlarge the dataset.

To increase the number of examples, we identified all of the proteins in SwissProt labeled as having alternatively spliced isoforms, giving 558 proteins with 1,266 regions that are absent from one isoform due to alternative splicing. Next, we predicted disorder/order for these alternatively spliced proteins and regions. As a control, we also predicted disorder/order for the 46 structurally characterized proteins and for their 75 regions that are affected by alternative splicing. For both datasets, we plotted the frequency of observation versus per cent disorder, with the disorder binned at the 20% level. The 75 alternatively spliced regions of known structure gave almost perfect agreement between predictions and observations. For the 1,266 regions from SwissProt, the predicted disorder closely matched the corresponding predictions for the 75 with known structure. These data strongly suggest that alternative splicing occurs mostly in regions of RNA that code for disordered protein.

Our previous predictions estimated that about 50% of mammalian proteins have disordered regions of 30 residues or longer [94]. These prediction results are similar to the estimate of 40% to 60% of mammalian genes that undergo alternative splicing. Thus, the overall likely frequency of intrinsic disorder is certainly high enough for 80% of alternative splicing events to occur in such regions.

In several sections given above, the various roles of disorder in protein functions and in protein-protein interaction networks are discussed. Modification of such functions including protein-protein interaction networks could be readily accomplished by alternative splicing within disordered regions. Thus, a linkage between alternative splicing and signaling by disordered regions provides a novel and plausible mechanism for understanding the origins of cell differentiation, which ultimately gave rise to multicellular organisms in nature [64]. New studies are needed to test these ideas.

## Conservation of intrinsic disorder in protein domains and families

Many proteins possess complex domain structure. In fact, ~65% proteins in unicellular organisms and > 80% proteins in metazoa, are multidomain proteins [162]. Traditionally, a domain is considered to be an independent (or semi-independent) part of a protein molecule that could fold autonomously, i.e., separately from the rest of the protein chain. Structural domains vary in length from between about 25 amino acids up to 500 amino acids in length. For structured proteins, domains have been described as units of independent folding [163], of compact structure [164] or of function and evolution [165]. Obviously, these definitions, being valid individually, may overlap and a conserved, compact structural domain is likely to be able to fold autonomously. Combinatorial usage of various structural and functional units creates a vast number of multidomain and multifunctional proteins [166], in which each domain may fulfill its own function independently, or in a concerted manner with its neighbors.

**Figure 9:**
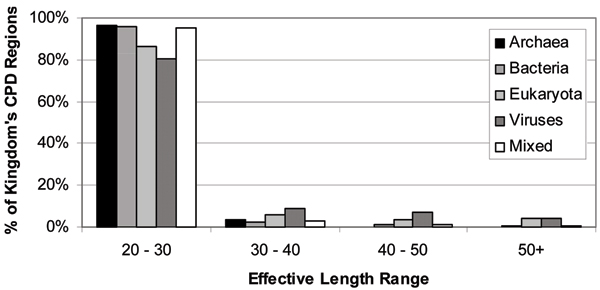
**Abundance of conserved predicted disordered regions in various organisms**. Histogram of conserved predicted disorder effective length classes by kingdom.

Recently [65,66], to identify the prevalence, characteristics, and functions of conserved disordered regions within protein domains and families, a database was created that stores the amino acid sequences of nearly one million proteins and their domain matches from the InterPro database [167]. InterPro is a computational resource integrating several protein family and domain databases, including PRINTS, PROSITE, Pfam, ProDom, SMART and TIGRFAMs [167]. These million proteins were analyzed using PONDR^® ^VL-XT disorder predictor and regions of sequence corresponding to domains were aligned using a multiple sequence alignment tool. Combining disorder prediction and conservation data, many regions of conserved predicted disorder were found within protein domains. This analysis identified 3653 regions of conserved disorder prediction, found within 2898 distinct InterPro entries [65]. Importantly, regions of conserved disorder prediction were found in protein domains from all available InterPro member databases. Furthermore, they were found in all kingdoms of life, including viruses [65]. Figure 9 emphasizes this fact and shows also that the majority of regions of conserved predicted disorder were short, with less than 10% of these regions founnd to exceed 30 residues in length. Most of the long conserved disordered regions were in domains from eukaryotic or viral proteins [65]. This is in line with our previous work, which found that long regions of intrinsic disorder were much more prevalent in eukaryotes than in prokaryotes [30,48,168]. This work has also shown that in addition to well-known conserved structural domains, protein domains and families have regions of conserved disorder. Most conserved disordered regions had sequence conservation greater than or equal to that in conserved ordered regions within the same protein. This indicated that disorder tendencies were kept in these proteins, suggesting that important functions likely depend on the disordered regions [65].

These regions of predicted disorder were found to be conserved within a large number of protein families and domains. Although many think of such conserved domains as being structured, in fact a significant number of them contain regions of disorder that are likely to be crucial to their functions [65]. The next crucial question was: What are the purposes of the regions of conserved predicted disorder? To answer this question, a functional repertoire of these regions was analyzed and a variety of functions were found to be associated with domains containing conserved disorder [66]. The most common were DNA/RNA binding and protein binding. Many ribosomal proteins also were found to contain conserved disordered regions. Other functions identified included membrane translocation and amino acid storage for germination. Due to limitations of both current knowledge and the methodology implemented in that study, it was not determined whether these functions were directly associated with the predicted disordered region [66]. Because in most cases the region of conserved predicted disorder covered only a part of the domain, it is possible that the disordered region is not required for the known function of the domain. However, given that this disorder is conserved through nearly all members of the domain, it seems likely that the disorder plays a role in at least one important function of the domain, whether that function is known or unknown. Furthermore, the functions associated with conserved disorder were in agreement with the functions found in other studies to correlate to disordered regions. We have established that intrinsic disorder may be more common in bacterial and archaeal proteins than previously thought, but this disorder is likely to be used for different purposes than in eukaryotic proteins, as well as occurring in shorter stretches of protein [66].

## The D^2 ^concept: abundance of untrinsic disorder in disease-related proteins

Proteins are crucial for life, so it is not surprising that their dysfunction can cause pathological conditions. Indeed, a significant number of diseases arise from the failure of a specific peptide or protein to adopt its proper structure. Such diseases are associated with protein misfolding. Observed consequences of misfolding include protein aggregation (and/or fibril formation), loss of normal function, and gain of toxic function. Some proteins exhibit a marked tendency to assume a pathologic conformation, and this tendency becomes increasingly evident with aging or at persistently high concentrations caused by some condition. Sometimes endogenous factors, including for example chaperones, intracellular or extracellular matrixes, other proteins and small molecules, can alter the conformation of a pathogenic protein and thereby increase its propensity to misfold. Other causes of misfolding and misfunction include point mutation(s), exposure to internal or external toxins, impaired posttranslational modifications (phosphorylation, advanced glycation, deamidation, racemization, acetylation, etc.), an increased probability of degradation, impaired trafficking, loss of binding partners or oxidative damage. These various factors can act independently or in complex associations. Intrinsically disordered proteins known as "hubs" associate with large numbers of partners (see above). Furthermore, such proteins often exhibit significant structural variability, forming different monomeric, oligomeric and insoluble conformations depending on the environment and suggesting that some of these proteins fold in a template-dependent manner (e.g., see [68]). From these observations, we proposed that the development of conformational diseases may originate, not only from misfolding, but also from misidentification, misregulation and missignaling [73]. That is, mutations and/or changes in the environment could cause protein confusion, thereby reducing the ability to recognize appropriate binding partners and leading instead to the occurrence of deadly aggregates.

**Figure 10:**
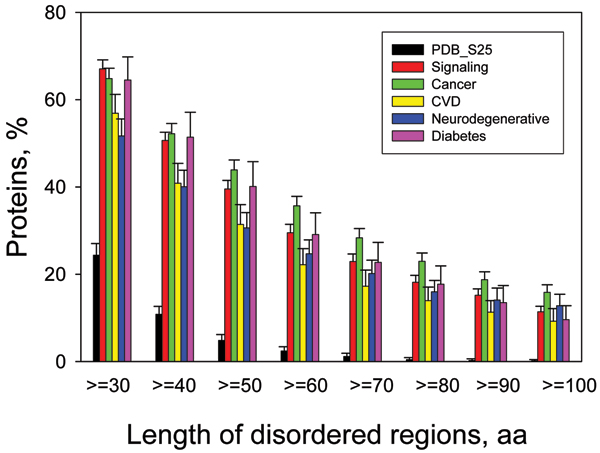
**Abundance of intrinsic disorder in disease-associated proteins**. Percentages of disease associated proteins with ≥ 30 to ≥ 100 consecutive residues predicted to be disordered. The error bars represent 95% confidence intervals and were calculated using 1,000 bootstrap re-sampling. Corresponding data for signaling and ordered proteins are shown for the comparison. Analyzed sets of diseas-related proteins included 1786, 487, 689, and 285 proteins for cancer, CVD, neurodegenerative disease and diabetes, respectively.

Data scattered in literature for individual proteins unambiguously show that some proteins involved in human diseases such as cancer, Parkinson's disease and other synucleinopathies, Alzheimer's, prion diseases, diabetes, and cardiovascular disease are either completely disordered or contain long disordered regions. This immediately raises the question of how abundant are such proteins in various pathological conditions. To answer this question, several sets of proteins related to various diseases, including cancer and cardiovascular disease (CVD), were collected and analyzed using a number of disorder predictors [67,70,71]. Results of these analyses are systemized in Figure 10, which shows percentages of proteins with ≥ 30 consecutive residues predicted to be disordered in datasets of proteins associated with cancer, CVD, neurodegenerative disease and diabetes [73]. This illustrates that intrinsic disorder is highly prevalent in CVD-, diabetes-, cancer, and neurodegenerative disease-related proteins, being comparable with that of signaling proteins and significantly exceeds the level of intrinsic disorder in eukaryotic proteins from SWISS-PROT and in non-homologous, structured proteins from the PDB. In fact, 79% of cancer-associated and 61% of CVD-associated proteins were found to contain predicted regions of disorder of 30 residues or longer [67,70,71,73].

Using CVD as an illustrative example, the hypothesis that high level of intrinsic disorder could be important for function of disease-related proteins, and for the control and regulation of processes associated with cardiovascular disease was confirmed by finding that 198 α-MoRFs were predicted in 101 proteins from the CVD dataset [71]. The mentioned number of MoRFs is important because these features provide the starting point for disorder-based drug discovery (see below). A comparison of disorder predictions with the experimental structural and functional data for a subset of the CVD-associated proteins indicated good agreement between predictions and observations.

Additional confirmation of the high prevalence of intrinsically disordered proteins in human diseases came from the described above functional annotation over the entire Swiss Protein database from a structured-versus-disordered point of view [55-57]. In fact, this analysis revealed that many diseases are strongly correlated with proteins predicted to be disordered. Contrary to this, we did not find disease-associated proteins to be strongly correlated with absence of disorder [57]. Among disease-related Swiss-Prot keywords strongly associated with intrinsic disorder were oncoproteins, malaria, trypanosomiasis, human immunodeficiency virus (HIV) and acquired immunodeficiency syndrome (AIDS), deafness, obesity, cardiovascular disease, diabetes mellitus, albinism, and prion [57]. Thus, intrinsic disorder is very common in disease-associated proteins, giving rise to the disorder in disorders concept, which we are calling the "D^2 ^concept." [73].

This high abundance of intrinsic disorder in proteins involved in various diseases suggests that they possess a number of specific features that make them key players in the development of pathological conditions. Intrinsically disordered regions or entire proteins are among major cellular regulators, recognizers and signal transducers. Their functionality is modulated via a number of posttranslational modifications and also can be tuned and made organ/tissue specific via the alternative splicing of corresponding mRNAs. Many intrinsically disordered regions and intrinsically disordered proteins can fold (completely or partially) upon interaction with corresponding binding partners, ensuring low-affinity/high-specificity binding. They possess multiple binding specificity and they are able to participate in one-to-many and many-to-one interactions [73]. All this makes intrinsically disordered regions and intrinsically disordered proteins very attractive targets for the development of a novel class of drugs aiming modulation of protein-protein interactions.

## Intrinsic disorder and drug discovery

For a long time protein-protein interactions have been a potential source of drug targets. Indeed, determining the protein interactome by systems biology approaches and understanding of these results at a deeper level points to interesting drug targets [169]. Despite such interest, developing drug molecules that block protein-protein interactions has not yet been successful [170,171]. Indeed, our searches of the current literature have failed to yield even one currently used drug molecule that functions by inhibiting a protein-protein interactions.

Even though there has been little success in finding drugs that act by blocking protein-protein interactions, several promising molecules are encouraging a renewed interest in this approach [172-175]. As pointed out in these recent discussions, several interesting drug-like lead compounds apparently function by blocking protein-protein interactions, and these leads are being actively pursued via drug-discovery strategies.

One very important interaction of interest, specifically the p53/Mdm2 interaction, has been the focus of multiple drug-discovery studies [176-178]. We became interested in this example because the binding region of p53 is intrinsically disordered [179]. However, the papers that discuss this interaction as a promising drug target don't even mention the disorder-to-order transition for the p53 partner.

Bioinformatics and computational structural biology tools were employed to investigate this interaction, and these studies revealed several features that explained why this region is so promising as a drug target. Next, we searched for similar features in other proteins contained in the human proteome. By this approach we found thousands of possible new drug targets involving one disordered partner. Many examples of these new targets are found for each of the major diseases [75]. Clearly a great deal of work is needed to find actual drug molecules based on these bioinformatics studies, but in our view these new leads merit systematic study.

A protein-protein interaction involving one disordered partner and one structured partner has several features that are consistent with being a good target for drug discovery. First, unlike most interfaces between two structured proteins, the interface between one structured and one disordered partner is almost never flat. Usually the structured partner has a cleft in the interface, while the disordered region typically becomes organized into a helix or other structure with hydrophobic side chains that project away from the backbone and into the cleft. Such features occur over and over in our MoRF dataset [59] and also in the examples used to develop the MoRF predictors [58,61]. With respect to the p53/Mdm2 interaction, the p53 binding site is predicted to be an α-MoRF, and this binding site contains hydrophobic side chains that project deeply into the cleft located on the surface of the Mdm2 partner.

For these disorder-based interactions, the disordered partner "morphs" from disorder-to-order, and, therefore, some of the binding energy must be spent to overcome the higher entropy of the unfolded state. Such an interaction is therefore likely to be weaker than a similar-sized interaction between two structured proteins. This entropy penalty means that such interactions will likely be easier to block with a small molecule competitor as compared to a similar interaction between two structured proteins.

**Figure 11:**
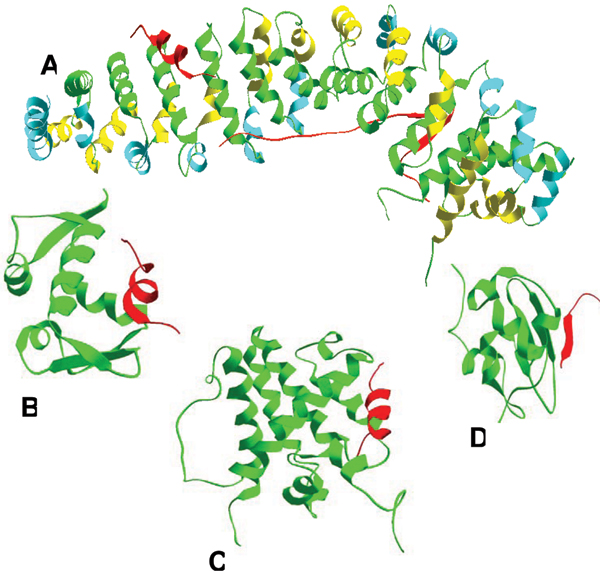
**IDPs as drug targets**. Protein-protein interactions involving α-helical or β-strand portion of the partners are used to design small molecules for cancer drugs. **A**. A ribbon diagram of complex of β-catenin (light colors) and T cell factor (red) was regenerated from PDB 1G3J. The structure of β-catenin is consisted of 12 tri-helical repeats (except the repeat 7, which just has two helical units). Small molecules from a natural-product library were screened and a couple of inhibitors were found. However, the binding sites for the small molecule inhibitors were not clear. **B**. A ribbon diagram of complex of MDM2 (green) and P53 fragment (red) was regenerated from PDB 1YCR. Small molecule inhibitors were designed based on the structure of the helical fragment of P53. **C**. A ribbon diagram of complex of Bcl-xL (green) and BAK fragment (red) was regenerated from PDB 1BXL. Small molecules were designed based on the 20-residue helix of BAK to inhibit the interaction. **D**. A ribbon diagram of complex of XIAP (green) and Smac fragment (red) was regenerated from PDB 1G3F. Small molecule inhibitors were designed based on the β-strand fragment (AVPIAQKSE) of Smac.

Protein disorder is not discussed or even mentioned in any of the papers touting protein-protein interactions as new targets for drug discovery. Nevertheless, 4 of the 8 examples described in these recent reviews [172,174] depend on one structured partner and one disordered partner. Furthermore, in 3 of the 4 examples, the disordered segments, or at least part of the disordered segment morphs into a helix upon binding (see Figure 11). Thus, the p53/Mdm2 complex is not alone in being a disorder-based interaction that is blocked by a small drug-like molecule. Many more examples are likely to appear in the coming years, and we anticipate that some of these examples will eventually lead to new drug molecules.

## Conclusion

The concepts regarding drug discovery can be linked with the concepts regarding alternative splicing. Together, these two concepts suggest approaches that could lead to the development of tissue-specific drugs via taking into account tissue-specific alternative splicing in disordered regions that form protein-protein interactions that can be blocked by small molecules. Such tissue-specific drug molecules might have fewer side effects than current drug molecules.

The concepts regarding drug discovery can be linked with the concepts regarding protein-protein interactions. Together these two concepts suggest two distinct possibilities. First is the possibility of one drug molecule blocking one protein-protein interaction (for one-to-many signaling interactions). Second is the possibility of one drug molecule blocking many interactions (for many-to-one signaling interactions). Experiments could be designed to focus specifically on these distinct classes of interactions to determine possible differential biological effects of these two distinct possibilities.

The concepts of drug discovery can be linked with the concepts regarding the functions of intrinsically disordered regions. Together these concepts suggest new strategies for finding drugs aimed at a wide variety of signaling and regulatory functions.

We began to apply bioinformatics to the set of disordered proteins about 12 years ago, with our first paper being published slightly more than 10 years ago [86]. During this decade our understanding of the biological functions and importance of these proteins has undergone a significant improvement. Perhaps the next decade will pave the way for practical outcomes (such as new drug molecules) from the study of these proteins.

## Competing interests

The authors declare that they have no competing interests.

## Authors' contributions

CJO, VV, and JWC have done the computational analysis, designed figures and contributed to the manuscript writing. JM and JYY were involved in finding and analysis of p53 and 14-3-3 binding partners. VNU was involved in planning of experiments, contributed to the manuscript writing and revised the final version. AKD was involved in design and planning of all the experiments, drafted the manuscript and headed the project. All authors have read and approved the final manuscript.
